# Maternal Satisfaction on Delivery Service and Its Associated Factors in Public Health Facilities at West Shewa Zone, Ethiopia

**DOI:** 10.4314/ejhs.v33i2.15

**Published:** 2023-03

**Authors:** Alemayehu Siffir Argawu, Maru Mossisa Erana

**Affiliations:** 1 Department of Statistics, College of Natural and Computational Sciences, Ambo University, Ethiopia; 2 Department of Midwifery, College of Medicine and Health Sciences, Ambo University, Ethiopia

**Keywords:** Maternal Satisfaction, Delivery Services, Health Institutions

## Abstract

**Background:**

Maternal delivery satisfaction could be assessed to improve the quality and effectiveness. Thus, this study aimed to assess maternal satisfaction with delivery services and associated factors in public health facilities in West Shewa Zone, Ethiopia.

**Methods:**

An institution-based cross-sectional study was conducted on maternal satisfaction delivery services. Systematic and stratified sampling techniques were used to select mothers by using their delivery registration number and to select health facilities. A binary logistic regression model was used to identify associated factors.

**Results:**

Among mothers, the overall satisfaction with delivery service was 584 (87%). Shower service availability (39.8%), toilet cleanliness (32.6%), and waiting area cleanliness (31.1%) were significant areas of mothers' dissatisfaction with delivery services. Uneducated mothers, mothers of 1–6 education level, monthly income of mothers less than 500 Birr, mothers who followed antenatal care, mothers who had actual fetal outcome, mothers who admitted from 6:00 AM to 12:00, and mothers who admitted from 12:00 AM to 6:00 PM were satisfied with delivery services.

**Conclusion:**

The age of the mother, mother's education level, monthly income of mother, antenatal care, fetal outcome, place of delivery, and admission time of the mother were significantly associated factors for mothers' satisfaction with delivery services. Therefore, regional health bureaus and zonal health offices should work collaboratively on maternal care to improve maternal satisfaction during delivery.

## Introduction

The Sustainable Development Goals (SDG) 2030, reduce the global maternal mortality ratio to less than 70 per 100 000 live births. Women who remain healthy during pregnancy and after delivery are more likely to stay healthy later in life and have better birth outcomes, influencing infancy, childhood and adulthood. Therefore, the health and well-being of women matter to every person, society and country and are essential to achieving the SDGs. Action is necessary across sectors and settings to eliminate avoidable maternal and perinatal mortality and morbidity (1).

Maternal delivery satisfaction could be assessed to improve the quality and effectiveness of women's health cares as WHO recommends. Quality of delivery care is the degree to which maternal health services for individuals and populations increase the likelihood of timely and appropriate treatment to achieve the desired outcomes. The use of services and products result from the provision of care and women's experience of that care (1, 2).

Globally, an estimated 295,000 maternal deaths occurred in 2017, yielding an overall maternal mortality rate of 211 maternal deaths per 100 000 live births in 185 countries (1). Ethiopia's maternal and neonatal mortality rates also remained high, with a maternal mortality rate of 412 per 100,000 pregnant women and a neonatal mortality rate of 29 per 1000 newborns. Over half (54%) of the maternal deaths in Ethiopia occurred in health facilities, and (36.4%) of the deaths were due to delays in receiving appropriate care after reaching a health institution (3).

Maternal deaths in Ethiopia have decreased. According to the 2016 Ethiopian Demographic and Health Survey, 412 maternal deaths per 100,000 live births occurred (3). Health services and community health workers may have increased due to government promises. The Ministry of Health conducted a maternal death review in 2014 to reduce the nation's high maternal mortality rate. This review system encourages hospital assessment of all maternal fatalities and maternal near-miss (4). Impressive progress has been made, with half of the women giving birth in a facility, reducing the risk of death due to complications during delivery. However, ensuring timely arrival and service quality at facilities remains a challenge. These conditions are exacerbated as the health system struggles to meet the demand for routine quality healthcare and the frequent need to respond to drought, conflict or disease outbreaks, including COVID-19 (5).

Some demographic characteristics have been studied concerning satisfaction during delivery services. Another study in Brazil found no age-related difference in women's satisfaction with childbirth services. However, a study in Sweden found that younger women had more negative expectations about childbirth and experienced more pain and a lack of control during labor than older women (6, 7). Studies from developing countries show that the educational level of women in different studies and settings has demonstrated positive, negative or nil association with satisfaction during delivery services (8–10).

Numerous qualitative studies on Indian women's experiences and opinions about giving birth at a health facility show that they are not entirely satisfied with the delivery service, primarily because of the lengthy wait before they meet a healthcare provider, the limited opportunities they have to communicate with providers, the lack of their participation in decision-making, and the stern care providers (11–14); despite this, they accept childbirth services deemed to be “essential” for safe childbirth (15–17).

While the community's access to institutional delivery has improved, the assumption that accessibility is synonymous with quality of care, especially among policymakers, gives concern (18–20).

Regardless of the implementation of sympathetic, respectful and caring health services by the Ethiopian Federal Ministry of Health, maternal satisfaction with delivery services at public hospitals is poorly addressed. In Ethiopia, most women are not confident in the quality of public hospital services. Moreover, based on our Google findings, only one similar study was conducted in West Shewa Zone public health facilities. Still, the study did not include the essential socio-demography variables (like the mother's age, education level, and admission time to the health facility) (21). Furthermore, these variables are associated factors for maternal satisfaction with delivery services in this study. Therefore, this study tried to overcome these gaps using a Likert scale questionnaire through independent observation of structural and process-related predictors. Thus, we aimed to assess maternal satisfaction with delivery services and associated factors in public health facilities at West Shewa Zone, State of Oromia, Ethiopia.

Studying the effectiveness of health facility delivery services from the client's perspective will give providers, decision-makers, local planners, and other stakeholders systematic information to help them better understand how well the service is going. This study has two objectives: knowledge generation and raising the standard of service delivery.

Local planners and decision-making can use the findings of this study to improve the quality of health facilities' delivery services.

## Methods

**Study design and period**: An institutional-based cross-sectional study was conducted to assess mothers' satisfaction with delivery services at West Shewa Zone, State of Oromia, Ethiopia, health institutions. The study period was from April 10 to June 12, 2021.

**Sample size and sampling procedure**: The sample size formula for this study was developed by (22) with a finite population size by considering different assumptions given below: Z=2.33 is a value from the standard normal distribution table at 98% confidence level, P=0.608 proportion of mothers satisfaction on deliveries services from the similar study (23), *e*=0.04 (4%) is the margin of error to take a large sample size, and N=2,052 is the total population size of mothers were taken from the selected hospitals and health centers after reviewing the total number of deliveries attended in the previous quarter report in each health facilities. Finally, the required sample size was n=668 mothers after considering the 15% non-response rate. Then, a systematic sampling technique was used to select mothers using their delivery registration number from each selected stratum (public health facility). And stratified sampling was applied to select health facilities. Thus, there were seven selected public health facilities: four hospitals and three health centers.

**Variables in the study**: Maternal satisfaction with delivery service (satisfied or not satisfied) was the dependent variable of this study. The independent variables of this study were sociodemographic factors, including age, marital status, religion, education, occupation and level of income and obstetric factors, including the type of delivery, health condition after delivery, ANC follow-ups, duration of labor for final delivery, fetal outcome, knowledge on ANC service, and other related variables.

**Operational definitions**: If mothers' responses of “satisfied” or “very satisfied” on the questionnaires related to satisfaction with delivery services are greater than or equal to 75% (15 out of 20), then the overall satisfaction of mothers was classified as “satisfied”, and otherwise it was classified as “unsatisfied” (7, 24).

**Data collection procedure and quality assurance**: First, the questionnaire was prepared in English, translated into Afaan-Oromo and Amharic languages, and then translated back into English to ensure consistency. The data collectors and supervisors were given two days of training about the study's objective and each questionnaire component. The pretest was conducted on 34 respondents (5% of the sample) from Ambo general hospital. The questionnaires were filled out by the mothers/caretakers with the help of data collectors when mothers were in beds after the delivery process was completed. Then, the investigators and supervisors made on-the-spot checking and reviewed all the completed questionnaires to ensure that complete and consistent information was collected and immediate action was taken.

**Inclusion criteria**: Mothers who delivered at the selected health facilities in the study area from April 10, 2021, to June 12, 2021, before discharge. And mothers/caretakers aged 15 to 49 were included in the study.

**Exclusion criteria**: Mothers who delivered at the selected health facilities in the study area and came for postnatal care were excluded to avoid recall bias. Moreover, mothers who were severely ill and unable to hear were excluded from the study.

**Data processing and analysis**: Data were entered and analyzed by using SPSS version 25. Descriptive statistics and charts were computed for the study variables. A binary logistic regression model was fitted to identify factors associated with mothers' satisfaction with delivery service, and a 5% significance level was used in the model analyses. The odds ratio and 95% confidence interval were used to determine the strength of the association between dependent and independent variables.

**Ethical consideration**: Ethical clearance letter was obtained from the Ambo University Research and Community Service Vice President's Office. Communication with each health institution administrator was made through a formal letter from the University. After the purpose and objective of the study were informed, verbal consent was obtained from each participant. Participants were also informed that participation would be voluntary and that they could withdraw from the study at any time. The confidentiality of information provided by study subjects was also protected by making the data collection procedure anonymous.

## Results

**Figure 1:**
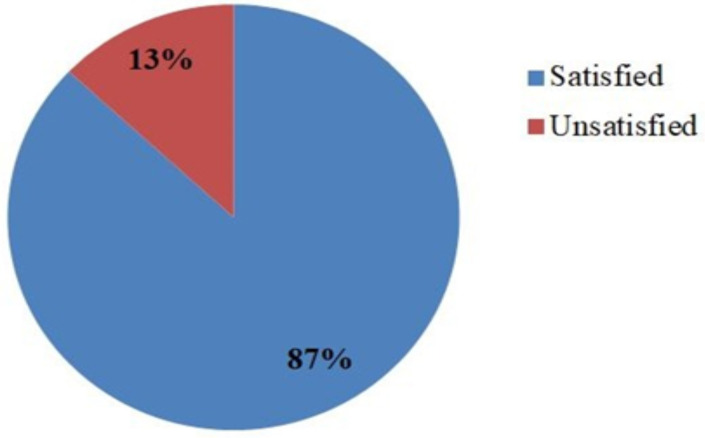
Prevalence of maternal satisfaction on delivery service in public health facilities at Westshewa Zone, state of Oromia, Ethiopia, 2021.

**Prevalence of maternal satisfaction with delivery service**: Among 668 mothers who delivered in public health facilities at West Shewa Zone, their overall satisfaction with delivery service was found to be 87% of them (Figure 1).

**Figure 2:**
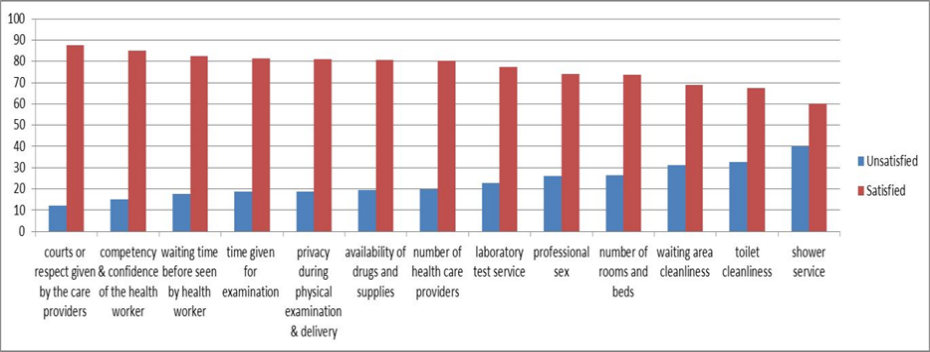
Bar charts for items comparison on maternal satisfaction levels in Westshewa Zone health institutions, state of Oromia, Ethiopia, 2021.

**Items comparison on maternal satisfaction levels**: The items comparisons on maternal satisfaction on delivery services were presented. Thus, courtesy and respect of the health care providers (87.7%), competency and confidence of the health workers (85.0%) and waiting time before being seen by health workers (82.3%) were the top three significant areas that mothers were satisfied. At the same time, shower service availability (39.8%), toilet cleanliness (32.6%) and waiting area cleanliness (31.1%) were the top three major areas that make mothers dissatisfied with delivery services (Figure 2).

**Factors associated with maternal satisfaction on delivery service:** In the multivariate logistic regression analysis, the age of the mother, educational status, monthly income, ANC follow-up, fetal outcome, health institution type, and admission time of the mother were significant factors for the overall maternal satisfaction on delivery services 5% level of significance (Table 1).

**Table 1:** Associated factors to mothers' delivery services satisfaction in univariate and multivariate analysis in Westshewa Zone health institutions, state of Oromia, Ethiopia, 2021

Variable	Satisfied	Unsatisfied	AOR (95% CI)
**Age of mother**			
< 20 years	25(64.1%)	14(35.9%)	0.28 (0.09, 0.83) *
20 – 34 years	405(88.6%)	52(11.4%)	1.06 (0.50, 2.22)
35 – 44 years	154(89.5%)	18(10.5%)	1
**Educational Status**			
No formal education	128(98.5%)	2(1.5%)	18.60 (3.22, 107.5) *
Grade 1 – 6	118(94.4%)	7(5.6%)	5.40 (1.76, 16.57) *
Grade 7 – 12	124(81.6%)	28(18.4%)	1.42 (0.65, 3.08)
Diploma & above	214(82.0%)	47(18.0%)	1
**Occupational status**			
Housewife	223(89.6%)	26(10.4%)	1.11 (0.41, 3.02)
Employee	157(80.9%)	37(19.1%)	1.18 (0.39, 3.49
Merchant	119(91.5%)	11(8.5%)	2.60 (0.80, 8.39)
Other	85(89.5%)	10(10.5%)	1
**Monthly income (ETB)**			
< 500	119(96.0%)	5(4.0%)	10.78 (2.75, 42.27) *
500 – 1000	131(88.5%)	17(11.5%)	0.91 (0.38, 2.18)
> 1000	334(84.3)	62(15.7%)	1
**ANC follow up**			
Yes	518(89.8%)	59(10.2%)	3.01 (1.27, 7.14) *
No	66(72.5%)	25(27.5%)	1
**Mode of delivery**			
Normal delivery	505(89.9%)	57(10.1%)	1.17 (0.24, 5.74)
Assisted delivery	58(74.4%)	20(25.6%)	0.95 (0.18, 5.03)
Caesarian section	21(75.0%)	7(25.0%)	1
**Fetal outcome**			
Live	567(89.6%)	66(10.4%)	4.32 (1.40, 13.30) *
Died	17(48.6%)	18(51.4%)	1
**Maternal outcome**			
Normal	544(89.8%)	62(10.2%)	1.39 (0.49, 4.01)
With complication	40(64.5%)	22(35.5%)	1
**Admission time**			
Morning-Midday (6am–12am)	143(94.7%)	8(5.3 %)	6.22 (2.36, 16.38) *
Midday-Evening (12 AM–6 PM)	174(96.7%)	6(3.3%)	10.29 (3.46, 30.61) *
Evening-Midnight(6pm–12pm)	177(80.8%)	42(19.2%)	1.56 (0.79, 3.08)
Midnight-Morning(12pm–6am)	90(76.3%)	28(23.7%)	1
**Health institution type**			
Hospital	307(83.2%)	62(16.8%)	0.05 (0.02, 0.20) *
Health center	277(92.6%)	22(7.4%)	1

* Significantly associated factors at p < 0.05

**Sociodemographic factors**: Mothers aged less than 20 had less satisfaction with delivery care than those aged 35–44 [p=0.022, AOR=0.28 and 95% CI (0.09, 0.83)]. Concerning mother's educational status, uneducated and grade 1–6 mothers were eighteen and five times more satisfied than mothers whose educational status was diploma and above [p=0.001, AOR=18.60 & 95% CI (3.22, 107.47)] and [p=0.003, AOR=5.40 & 95% CI (1.76, 16.57)], respectively. Regarding the economic status of delivering mothers, the odds of satisfaction for the mothers whose monthly income was less than 500 ETB were ten times more satisfied than those whose income was higher than 1000 ETB [p=0.001, AOR=10.78 & 95% CI (2.75, 42.27)].

**Obstetrics history factors**: The participants who had ANC follow-up were found to have threefold increased odds of maternal satisfaction compared to those who had no ANC follow-up [p=0.013, AOR = 3.01 & 95% CI (1.27, 7.14)]. Mothers' fetal outcome was also a significant factor in maternal satisfaction. Mothers whose fetal outcomes were lived had four times more satisfaction than those whose fetal outcomes were died [p=0.011, AOR = 4.32 and 95% CI (1.40, 13.30)].

**Healthcare facilities factors**: Based on the admission time of mothers to the health facility, mothers who had been admitted in the health institutions from morning to mid-day (6:00 AM – 12:00 AM) and mid-day to evening (12:00 AM – 6:00 PM) had 6 and 10 times more satisfied than those who admitted from midnight to morning (12:00 PM – 12:00 AM) [p=0.000, AOR=6.22 & 95% CI (2.36, 16.38)] and [p=0.000, AOR=10.29 & 95% CI (3.46, 30.61)], respectively. According to health institution type, mothers delivered in the hospital were less satisfied with delivery service than those delivered in the health center [p=0.000, AOR=0.05 & 95% CI (0.02, 0.20)].

## Discussion

This study revealed that the overall proportion of maternal satisfaction with delivery services was 87%. It is in line with studies conducted in West Gojjam Zone at Bure Town at 88% (8), Ambo Town at 83.9% (25) in Ethiopia, and in Kenya Kiambu public facility at 87% (26), and in Southern Mozambique 92.5% (27).

The overall proportion of maternal satisfaction with delivery service was higher than with various related studies conducted in Nekemte Town at 82% (28), Debre Markos Town at 81.7% (7), Assela Hospital at 80.7% (29), Gamo Gofa Zone 79.1% (30), Bahir Dar City 74.9% (31), Adama Town 74.8% (32), West Arsi 74.6% (33), Wolaita Sodo University of Teaching and Referral Hospital 67.3% (34), University of Gondar Teaching and Referral Hospital 65.5% and 47.6% (35), Jimma Zone at Omo Nada District 65.2% (10), Oromia West Shewa Zone 60.8% (23) in Ethiopia, upper Egypt 78.5% (36), and in Indian Chhattisgarh 68.7% (37).

These variations might be due to the quality of services provided in the health facilities, type of health facilities, mothers' differing expectations, study period, structural elements of the health facilities, process determinants (interpersonal behavior, privacy, promptness, cognitive care, perceived provider competency and emotional support), mothers' utilization of skilled delivery services, and also outcome related determinants like health status of the mother and newborn (9, 24, 26, 38).

The main areas of dissatisfaction in the current study were shower service availability, toilet cleanliness and waiting area cleanliness. It was in line with previous studies that indicated the overall cleanliness and comfort of examination rooms and waiting areas, cleanliness and access to the toilets, and availability of hand-washing facility and shower were the main areas of dissatisfaction (8, 23, 30, 33).

Mothers aged less than 20 were less likely to be satisfied with delivery services than those aged 35–44 [AOR=0.28 & 95% CI (0.09, 0.83)]. It indicates that older mothers had more satisfied than younger and middle age groups. This finding is supported by other studies conducted in Bahir Dar City and the University of Gondar Referral Hospital(31, 35). This might be related to the fact that as the mother's age increases, the stability of her life situation increases, and she is more prepared for medical and psychological stresses. It differed from those studies conducted in Adama Town, Assela Hospital, Wolaita Zone and Jimma Zone (10, 29, 32).

Concerning mother's educational status, uneducated and grade 1–6 mothers were eighteen and five times more satisfied than mothers whose educational status was diploma and above [AOR=18.60 & 95% CI (3.22, 107.47)] and [AOR=5.40 & 95% CI (1.76, 16.57)], respectively. It showed that less educated mothers tended to have higher satisfaction than mothers above the sixth grade. It could be because educated mothers have high expectations for the services provided by delivery services. The finding is consistent with other studies from Ethiopia (29, 32, 34), and Egypt (36). This finding contradicts a study from the West Arsi Zone in Ethiopia (33).

The economic status of delivering mothers, mothers whose monthly income was less than 500 ETB, was eleven times more satisfied than those whose income was higher than 1000 ETB [AOR=10.78 & 95% CI (2.75, 42.27)]. It might be due to the difference in economic status that was delivering mothers whose monthly income was more incredible than 1000 ETB had the potential to be served wherever they wanted, like private clinics or hospitals. However, those delivering mothers whose income was less than 500 or 1000 ETB could not have the potential to afford the expense. It was consistent with other studies in Assela hospital and West Arsi from Ethiopia (29, 33). Moreover, another study from Sri Lanka also revealed that low-income groups had higher satisfaction with delivery services (39). This finding contradicts a study conducted in Ambo town general hospital in Ethiopia, which showed that women with low monthly incomes were less satisfied than women with high monthly incomes (25).

Our finding also revealed that ANC follow-up positively affected maternal satisfaction. Mothers who had their ANC follow-up were found to have threefold increased odds of maternal satisfaction compared to those who did not [AOR = 3.01 & 95% CI (1.27, 7.14)]. It is in line with different studies conducted in Ethiopia (10, 29, 33, 34). It implies that access to counselling sessions during prenatal visits about the benefits of institutional delivery may shape a harmonious bond in which providers and mothers become friendly during the delivery care process. In contrast to this study, the study done in Bahir Dar city showed that women who did not attend ANC were three times more likely to satisfy than women who attended ANC (31).

The fetal outcome was also a significant factor for maternal satisfaction; mothers whose fetal outcomes were four times more satisfied than those whose fetal outcomes were died [AOR = 4.32 & 95% CI (1.40, 13.30)]. The finding is agreed with studies from Ethiopia in West Arsi Zone and the University of Gondar Referral Hospital, and from Nigeria at UDUTH Sokoto showed that mothers whose fetal outcomes alive were more satisfied than mothers who had a fetal complication (33, 35).

The admission time of the mother to the health facility is another significant variable in this study. Mothers who were admitted from 6:00 AM –12 AM and 12:00 AM–6:00 PM were 6 and 10 times more satisfied than those who were admitted from 12:00 PM–6:00 AM [AOR=6.22 & 95% CI (2.36, 16.38)] and [AOR=10.29 & 95% CI (3.46, 30.61)], respectively. It might be because more care providers were available in the daytime than at night. This finding is consistent with the study done in Addis Ababa city and Jimma town public health centers that mothers who delivered in day time more satisfied than those who gave birth during the nighttime (10).

Another important risk factor is health institution type, mothers delivered in the hospital were less likely to be satisfied than those delivered in a health center [AOR=0.05 & 95% CI (0.02, 0.20)]. This finding is supported by studies conducted in Gamo Gofa Zone from Ethiopia that the proportion of the women attending health centers was more satisfied than women attending hospitals (30). In Southern Mozambique, mothers who gave birth in primary-level facilities tended to be more satisfied than those who gave birth in hospitals (27). This satisfaction might be due to the proximity of the health facility, and mothers who live a long distance from health facilities are more likely to delay reaching the hospitals on time (40). Another reason is that hospitals provide more services to patients/clients than health centers.

Due to security and peace issues in several areas of the study region, some data were not collected at the scheduled time. In addition, some responders did not initially want to answer the questions, but after hours of discussions, they did. Another drawback is that because study participants find it difficult to express their displeasure in front of data collectors, the social desirability bias may have negatively impacted the quality of the information gathered. Despite this, non-staff midwives collected the data privately to minimize bias.

In conclusion, the finding of this study indicated that 87.4% of mothers involved in the study were satisfied with delivery services in health institutions. Courtesy and respect by the health care providers, competency and confidence of the health workers and waiting time before being seen by health workers were the top three main indicators of mothers' dissatisfaction. At the same time, shower service availability, toilet cleanliness and waiting area cleanliness were major areas of dissatisfaction with mothers' delivery services. Moreover, the mother's age, educational status, monthly income, ANC follow-up, fetal outcome, admission time, and health institution type were significantly associated factors for maternal satisfaction in delivery services. Thus, it needs to take interventions and improvement on these indicators and associated factors. Moreover, evaluating health care services uptake from the client's point of view and targeting to identify problems is very necessary.

## References

[1] World Health Organization (2019). Trends in maternal mortality 2000 to 2017: estimates by WHO, UNICEF, UNFPA, World Bank Group and the United Nations Population Division.

[2] Van den Broek N, Graham W (2009). Quality of care for maternal and newborn health: the neglected agenda. BJOG.

[3] Central Statistical Agency (CSA) [Ethiopia] and ICF International (2016). Ethiopia Demographic and Health Survey 2016.

[4] Federal Democratic Republic of Ethiopia Ministry of Health (2012). Maternal death surveillance and response (MDSR) technical guideline.

[5] USAID (2020). <Ethiopia-Fact-Sheet_Maternal-Child-Health Oct-2020.pdf>.

[6] Sawyer A, Ayers S, Abbott J, Gyte G, Rabe H, Duley L (2013). Measures of satisfaction with care during labour and birth: a comparative review. BMC pregnancy and childbirth.

[7] Bitew K, Ayichiluhm M, Yimam K (2015). Maternal satisfaction on delivery service and its associated factors among mothers who gave birth in public health facilities of Debre Markos town, Northwest Ethiopia. BioMed research international.

[8] Asres GD (2018). Satisfaction and associated factors among mothers delivered at Asrade Zewude memorial primary hospital, Bure, west Gojjam, Amhara, Ethiopia: a cross sectional study. Primary Health Care: Open Access.

[9] Srivastava A, Avan BI, Rajbangshi P, Bhattacharyya S (2015). Determinants of women's satisfaction with maternal health care: a review of literature from developing countries. BMC pregnancy and childbirth.

[10] Tadesse BH, Bayou NB, Nebeb GT (2017). Mothers' satisfaction with institutional delivery service in public health facilities of Omo Nada District, Jimma Zone. Clinical Medicine Research.

[11] Melese T, Gebrehiwot Y, Bisetegne D, Habte D (2014). Assessment of client satisfaction in labor and delivery services at a maternity referral hospital in Ethiopia. The Pan African Medical Journal.

[12] Christiaens W, Bracke P (2007). Assessment of social psychological determinants of satisfaction with childbirth in a cross-national perspective. BMC pregnancy and childbirth.

[13] Bhattacharyya S, Srivastava A, Roy R, Avan BI (2016). Factors influencing women's preference for health facility deliveries in Jharkhand state, India: a cross sectional analysis. BMC Pregnancy and childbirth.

[14] Camacho FT, Weisman CS, Anderson RT, Hillemeier MM, Schaefer EW, Paul IM (2012). Development and validation of a scale measuring satisfaction with maternal and newborn health care following childbirth. Maternal and child health journal.

[15] Mukhopadhyay DK, Mukhopadhyay S, Mallik S, Nayak S, Biswas AK, Biswas AB (2016). A study on utilization of Janani Suraksha Yojana and its association with institutional delivery in the state of West Bengal, India. Indian journal of public health.

[16] Sabde Y, De Costa A, Diwan V (2014). A spatial analysis to study access to emergency obstetric transport services under the public private “Janani Express Yojana” program in two districts of Madhya Pradesh, India. Reproductive Health.

[17] Das P, Basu M, Tikadar T, Biswas G, Mridha P, Pal R (2010). Client satisfaction on maternal and child health services in rural Bengal. Indian journal of community medicin.

[18] Teferra AS, Alemu FM, Woldeyohannes SM (2012). Institutional delivery service utilization and associated factors among mothers who gave birth in the last 12 months in Sekela District, North West of Ethiopia: A community-based cross sectional study. BMC pregnancy and childbirth.

[19] Kesterton AJ, Cleland J, Sloggett A, Ronsmans C (2010). Institutional delivery in rural India: the relative importance of accessibility and economic status. BMC pregnancy and childbirth.

[20] Saha R, Paul P (2021). Institutional deliveries in India's nine low performing states: levels, determinants and accessibility. Global Health Action.

[21] Bulto GA, Demissie DB, Tasu TL, Demisse GA (2020). Mother's satisfaction with the existing labor and delivery care services at public health facilities in West Shewa zone, Oromia region, Ethiopia. BMC pregnancy and childbirth.

[22] Kothari CR (2004). Research methodology: Methods and techniques: New Age International.

[23] Bulto GA, Demissie DB, Tasu TL, Demisse GA (2020). Mother's satisfaction with the existing labor and delivery care services at public health facilities in West Shewa zone, Oromia region, Ethiopia. BMC pregnancy and childbirth.

[24] Tayelgn A, Zegeye DT, Kebede Y (2011). Mothers' satisfaction with referral hospital delivery service in Amhara Region, Ethiopia. BMC pregnancy and childbirth.

[25] Gejea T, Abadiga M, Hasen T (2020). Maternal Satisfaction with Delivery Services of Government Hospitals in Ambo Town, West Shoa Zone, Oromia Region, Ethiopia, 2020. Patient preference and adherence.

[26] Okumu C, Oyugi B (2018). Clients' satisfaction with quality of childbirth services: A comparative study between public and private facilities in Limuru Sub-County, Kiambu, Kenya. PloS one.

[27] Mocumbi S, Högberg U, Lampa E, Sacoor C, Valá A, Bergström A (2019). Mothers' satisfaction with care during facility-based childbirth: a cross-sectional survey in southern Mozambique. BMC pregnancy and childbirth.

[28] Babure Z, Assefa J, Weldemarium T (2020). Maternal Satisfaction and Associated Factors towards Delivery Service among Mothers sectional Study Design. Women's Health Care.

[29] Amdemichael R, Tafa M, Fekadu H (2014). Maternal satisfaction with the delivery services in Assela Hospital, Arsi zone, Oromia region. Gynecol Obstet (Sunnyvale).

[30] Tesfaye R, Worku A, Godana W, Lindtjorn B (2016). Client satisfaction with delivery care service and associated factors in the public health facilities of Gamo Gofa zone, Southwest Ethiopia: in a resource limited setting. Obstetrics and gynecology international.

[31] Mekonnen ME, Yalew WA, Anteneh ZA (2015). Women's satisfaction with childbirth care in Felege Hiwot Referral Hospital, Bahir Dar city, Northwest Ethiopia, 2014: cross sectional study. BMC research notes.

[32] Tadele M, Bikila D, Fite RO, Obsa MS (2020). Maternal satisfaction towards childbirth Service in Public Health Facilities at Adama town, Ethiopia. Reproductive health.

[33] Urgessa A (2016). Mothers' Satisfation with Delivery Services and Associated Factor Sat Health Institutions in West ARSI, Oromia Regional State.

[34] Wolka S, Assegid S, Temesgen Tantu MG, Duko B (2020). Determinants of Maternal Satisfaction with Existing Delivery Care at Wolaita Sodo University Teaching and Referral Hospital, Ethiopia. BioMed Research International.

[35] Taddese AA, Gashaye KT, Dagne H, Andualem Z (2020). Maternal and partner's level of satisfaction on the delivery room service in University of Gondar Referral Hospital, northwest, Ethiopia: a comparative cross-sectional study. BMC health services research.

[36] Sayed W, Abd ElAal DEM, Mohammed HS, Abbas AM, Zahran KM (2018). Maternal satisfaction with delivery services at tertiary university hospital in upper Egypt, is it actually satisfying. Int J Reprod Contracept Obstet Gynecol.

[37] Jha P, Larsson M, Christensson K, Skoog Svanberg A (2017). Satisfaction with childbirth services provided in public health facilities: results from a cross-sectional survey among postnatal women in Chhattisgarh, India. Global health action.

[38] Tesfaw N, Gizachew A, Kassa GM, Abajobir AA (2018). Skilled delivery service utilization and associated factors among mothers who gave birth in the last two years in Northwest Ethiopia. Ethiopian Journal of Health Sciences.

[39] Senarath U, Fernando DN, Rodrigo I (2006). Factors determining client satisfaction with hospital-based perinatal care in Sri Lanka. Tropical Medicine & International Health.

[40] Awel S, Bagilkar VV, Fekecha BJEJoHS (2022). Delay in reaching institutional delivery service utilization among mothers attending Jimma Medical Center, Ethiopia. Ethiopian Journal of Health Sciences.

